# Case Report: Immunotherapy-induced Felty syndrome in a patient with metastatic melanoma

**DOI:** 10.3389/fonc.2026.1808710

**Published:** 2026-04-20

**Authors:** Laura S. Park, Caoimhe Byrne, Hayley Burridge, Wendy Zhu, Michelle Leech, Miles C. Andrews

**Affiliations:** 1 Medical Oncology, Alfred Health, Melbourne, VIC, Australia; 2 Department of Rheumatology, Monash Health, Clayton, VIC, Australia; 3 Faculty of Medicine Nursing and Health Sciences, Monash University, Clayton, VIC, Australia; 4 Department of Cancer Medicine, School of Translational Medicine, Monash University, Melbourne, VIC, Australia

**Keywords:** case report, Felty syndrome, immunotherapy, immune-related adverse events, melanoma, rheumatoid arthritis

## Abstract

Immune checkpoint inhibitors (ICIs) are critical in the modern treatment of advanced melanoma but can provoke immune-related adverse events (irAEs) with increased risk in patients having pre-existing autoimmune conditions. We present a case of Felty syndrome (FS) recurrence precipitated by a single dose of combination nivolumab plus relatlimab immunotherapy in a 71-year-old man with metastatic melanoma and a history of seropositive rheumatoid arthritis (RA) diagnosed several years earlier in the context of spontaneous FS. Following ICI, he presented with a neutrophil count of 0.0 cells/µL but without clinically active synovitis. He was promptly managed with intravenous methylprednisolone and methotrexate with rapid neutrophil recovery despite the ICI precipitant. In this report, we explore the differential diagnoses and treatment considerations, which highlight the importance of considering ICI-induced FS as a differential diagnosis in patients with a history of RA presenting with neutropenia. Early identification and collaborative care are vital for preventing life-threatening complications associated with neutropenia in this vulnerable patient population.

## Introduction

Immune checkpoint inhibitors (ICIs) are currently the mainstay of treatment for advanced or metastatic melanoma. Approved regimens include single or combination therapy of monoclonal antibodies targeting programmed cell death-1 (PD-1) receptor, cytotoxic T lymphocyte-associated protein 4 (CTLA-4), and lymphocyte activation gene 3 (LAG-3). ICIs improve overall survival in patients with metastatic melanoma but risk triggering immune-related adverse events (irAEs) that are diverse in type and severity. Patients with pre-existing autoimmune conditions like rheumatoid arthritis (RA) are at relatively high risk of recurrence of their conditions in addition to the standard risks of new irAEs (1). Felty syndrome (FS) is a rare but serious subset of seropositive RA with prominent extra-articular manifestations of splenomegaly and neutropenia. FS previously had a 5-year mortality of 36% with infections being the most common cause of death due to severe neutropenia (2). Prognosis has improved with advances in disease-modifying anti-rheumatic drugs (DMARDs) and wider availability of granulocyte colony-stimulating factor (G-CSF). We report a case of ICI-related FS in a patient with an index episode of FS 11 years previously, in whom rapid absolute neutrophil count (ANC) recovery was achieved with prednisolone and reinstitution of methotrexate, demonstrating the high responsiveness of FS to prompt initiation of moderate immunosuppression even in the context of ICI.

## Case description

**Figure 1:**
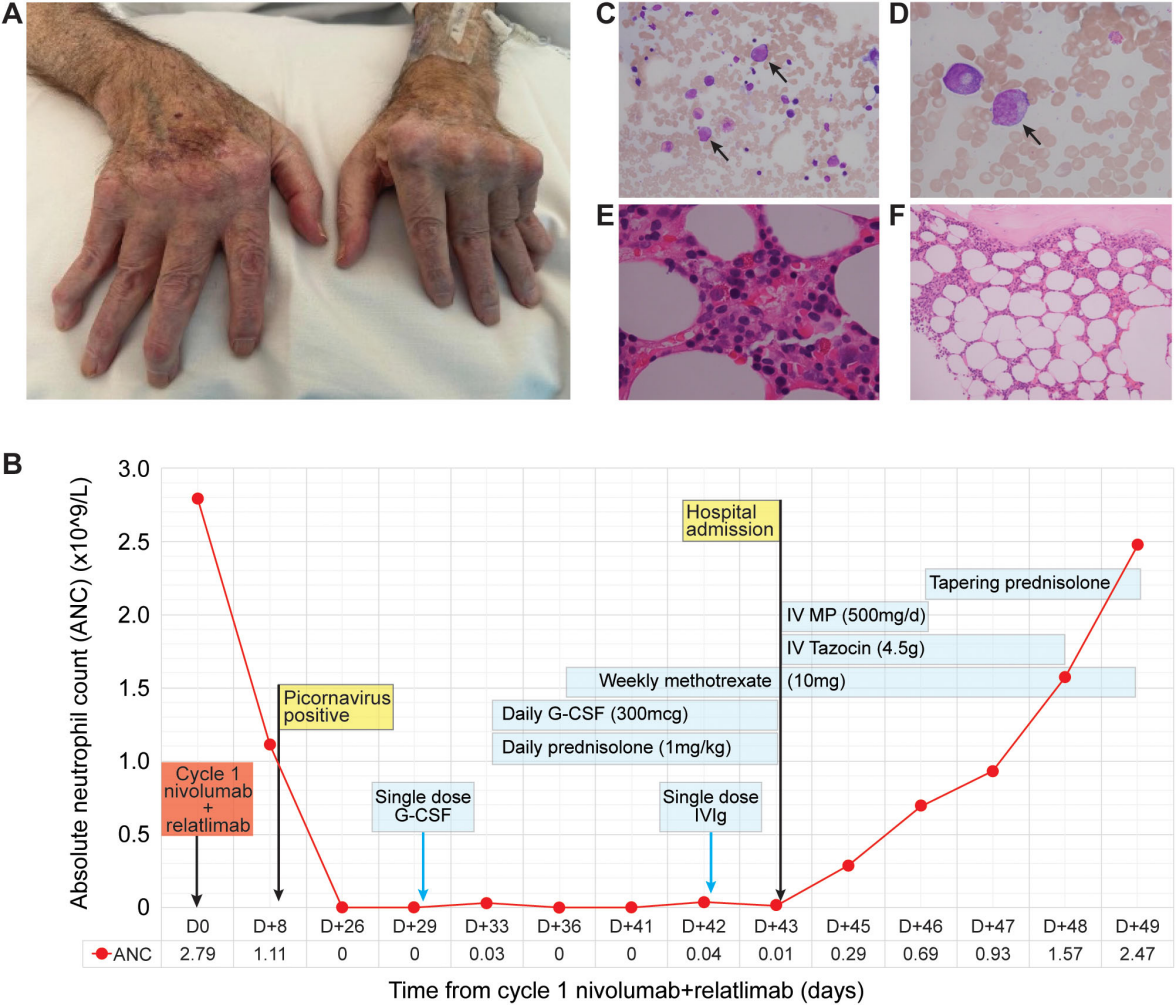
**(A)** Typical hand deformities of rheumatoid arthritis, including swan neck deformities, boutonniere deformity, ulnar deviation, subluxation of metacarpophalangeal joints, and Z-deformity. **(B)** Timeline of major clinical events and management following commencement of nivolumab + relatlimab. G-CSF, granulocyte colony-stimulating factor; IVIg, intravenous immunoglobulin; MP, methylprednisolone; Tazocin, piperacillin + tazobactam. **(C–F)** Bone marrow aspirate and trephine. Bright-field images of H&E-stained bone marrow aspirate at ×40 **(C)** and ×100 **(D)** magnification, and trephine sections at ×40 **(E)** and ×20 **(F)** magnification, showing granulocytic maturation limited to promyelocytes (arrows) with no myelocytes, metamyelocytes, or segmented neutrophils, but preserved erythropoiesis and megakaryopoiesis.

The patient was diagnosed with RA at 59 years of age when his hand deformities (**Figure 1A**) were incidentally noted along with a serum rheumatoid factor (RF) of 6,600 kU/L. With additional findings of splenomegaly and neutropenia, FS was suspected and subsequently confirmed on a bone marrow aspirate and trephine biopsy. He was started on methotrexate, but 1 month later, he presented with neutropenic multimicrobial septic shock (ANC 0/L) resulting in a prolonged admission. Neutrophil count recovery was ultimately achieved with G-CSF support, and methotrexate was recommenced.

Eleven years later, at the age of 70 years, the patient was diagnosed with stage IIIC (T4bN2aM0, AJCC 8th edition) mixed nodular/superficial spreading melanoma harboring a *BRAF* V600E mutation after a wide local excision of an ulcerated upper back lesion with 2 out of 3 positive sentinel lymph nodes and no distant metastatic disease on an FDG-PET/CT scan. Given this new high-risk melanoma diagnosis, the patient’s methotrexate (10 mg weekly) was weaned off in consultation with his usual rheumatologist. He was commenced on adjuvant targeted therapy with dabrafenib and trametinib, which were ceased 6 months later due to intolerable treatment-related fevers, weakness, and fatigue. The patient continued high-risk clinical surveillance, during which two new stage IIB primary nodular melanomas were excised from the left temple and right cheek, 4 months after ceasing adjuvant therapy. A follow-up FDG-PET/CT scan at 5 months after ceasing adjuvant therapy revealed distant metastatic melanoma recurrence involving the lung, pleura, and a malignant pleural effusion. Given the rapid development of metastatic disease post-adjuvant therapy, presumed to harbor an adversely prognostic *BRAF* V600E mutation, and symptomatic burden of disease, combination immunotherapy was deemed warranted. However, given the significant competing risk of RA flare, combination immunotherapy with nivolumab and relatlimab was commenced in preference to ipilimumab plus nivolumab, given the numerically lower documented risk of high-grade irAEs in clinical trials and lack of intracranial metastases for which the latter regimen is more strongly indicated (3, 4). Prior to commencing therapy, ANC was within normal limits.

One week following his first dose of nivolumab and relatlimab, the patient presented to the clinic with worsening exertional dyspnea, headaches, and rhinorrhea. A respiratory viral swab was positive for picornavirus, which was managed conservatively, and his known left-sided pleural effusion was drained. ANC was 1.11 × 10^9^/L.

Two weeks later, ANC was 0.0 × 10^9^/L. Besides his baseline dyspnea and mild bilateral hand arthralgia, the patient reported no new symptoms. Differential diagnoses included immunotherapy-triggered FS, post-viral agranulocytosis, or immunotherapy-related neutropenia. The hematology team’s consultation favored a diagnosis of post-picornaviral agranulocytosis considering the recent illness, clinical stability of RA, and lack of splenomegaly on clinical exam and imaging. The patient was given one dose of G-CSF with close clinical and blood monitoring. When his ANC remained at 0.0 × 10^9^/L, daily G-CSF was recommended for presumed nivolumab-induced neutropenia. The patient’s usual rheumatology team from an external hospital was consulted given elevated CRP at 122 mg/L with RF of 4,080 kU/L and anti-CCP antibodies of 643 units/mL (**Table 1**). Suspicious of immunotherapy-related FS, the patient was commenced on prednisolone 75 mg daily (1 mg/kg dosing) with ongoing daily G-CSF and *Pneumocystis* prophylaxis with oral trimethoprim/sulfamethoxazole 160 mg/800 mg three times weekly. No improvement in ANC was seen after 5 days (**Figure 1B**). Even with the reintroduction of methotrexate at 10 mg weekly, ANC remained <0.01 × 10^9^/L. Clinically, the patient had developed a new oral ulcer but was otherwise well. A dose of intravenous immunoglobulin (IVIg) was administered, and a bone marrow biopsy was scheduled.

**Table 1:** Blood test results on day 29 after the first dose of nivolumab + relatlimab.

Hemoglobin	122 g/L
Total white cell count	1.53 × 10^9^/L
Platelets	160 × 10^9^/L
Neutrophils	0.0 × 10^9^/L
Lymphocytes	1.02 × 10^9^/L
Blood film	Red cells show moderate rouleaux. No neutrophils seen on blood film. No blasts. Platelets appear normal.
C-reactive protein	122 mg/L
B_12_	249 pmol/L
Folate	17.7 nmol/L
Erythrocyte sedimentation rate	73 mm/h
Rheumatoid factor	4,080 kU/L
Anti-CCP antibodies	643 units/mL

On the day of the planned biopsy, 1 week after the first dose of methotrexate, the patient presented to the hospital with a 1-day history of diarrhea and abdominal pain. ANC was 0.01 × 10^9^/L and CRP was elevated at 117 mg/L. Abdominal CT scan demonstrated gastroenteritis, colitis, and uncomplicated diverticulitis. The stool sample was positive for *Clostridioides difficile* on culture but negative for toxin. He was admitted to the hospital for IV piperacillin–tazobactam and IV methylprednisolone 500 mg/day (3-day pulse) to treat his likely ICI-induced FS and neutropenic colitis. Daily G-CSF was ceased on admission, but weekly methotrexate was continued. Bone marrow biopsy showed normocellular marrow with granulocytic maturation arrest and no evidence of hematological or metastatic malignancy (**Figures 1C–F**). Flow cytometry revealed lymphocytosis (58% of nucleated cells, including 79% T cells with a CD4:8 ratio of 0.7:1) and an abnormal but non-clonal T-cell population (17%) being CD3^dim^CD5^dim^CD8^+^CD7^+/bright^CD2^dim^, TCR-CB1 polytypic. Collectively, these findings and the temporal relationship with initiation of ICI were most consistent with a form of immune-mediated neutropenia rather than chemical toxicity or malignant marrow infiltration. Given the patient’s history of FS and elevated RA markers, the diagnosis was deemed to be FS triggered by ICI. ANC improved to 0.69 × 10^9^/L on day 4. His diarrhea settled and antibiotics were de-escalated to oral amoxicillin–clavulanic acid. With continued clinical and ANC improvement, he was discharged home after 1 week on a slow-tapering regimen of oral prednisolone from 100 mg/day. Nivolumab and relatlimab for his melanoma were permanently discontinued, and no further immunotherapy was considered. The patient was not eligible for any available clinical trials. Repeat molecular testing of his pleural metastatic disease revealed an *NRAS* mutation and no *BRAF* mutation, indicating metastasis from a separate primary than his previous stage III disease, with no available targeted therapy. No further treatment was received, and the patient eventually died from disease progression approximately 6 months later.

## Discussion

FS is a clinical diagnosis that classically manifests with a triad of RA, splenomegaly, and neutropenia, although splenomegaly is not a requirement for diagnosis such as in this case. The pathogenesis of neutropenia in FS is multifactorial including failure of bone marrow to produce or mature neutrophils, autoantibodies against G-CSF, cell-mediated granulocyte destruction, and splenic sequestration of neutrophils (5). Management of FS involves DMARDs, G-CSF, steroids, and in refractory cases, splenectomy. Given a limited understanding of ICI-triggered FS, our management was guided by a combination of the treatment for FS and isolated hematological irAE. Despite methotrexate, G-CSF, IVIg, and steroids, an improvement in ANC in our case only occurred after pulsed IV methylprednisolone at least 1 week after reinstitution of methotrexate. In the event of pulsed steroid failure, a multidisciplinary decision had been made to trial the CTLA-4 agonist abatacept on the basis that an ICI-stimulated cytotoxic T-cell contribution was highly likely in this patient; thus, T-cell inhibition with abatacept was deemed a reasonable salvage medical therapeutic option. Multiple case reports demonstrate the efficacy of abatacept in other steroid-refractory high-fatality irAEs, particularly myocarditis with or without overlapping myasthenia and myositis (“triple M syndrome”) (6, 7), supporting the evaluation of abatacept in formal phase II and III clinical trials (e.g., NCT05195645, NCT05335928).

This case highlights the complexity of management decisions in ICI-treated patients presenting with complications of unclear and multifactorial etiology. Viral agents are a well-known cause of isolated neutropenia but an unlikely cause in this case when the ANC did not improve with G-CSF. In a review of 20 published cases, isolated ICI-induced neutropenia demonstrated no sex preponderance, was observed in a wide range of age groups most commonly following anti-PD-1 therapy for lung cancer or melanoma (8), and was variably managed with steroids, IVIg, G-CSF, and/or immunosuppressants such as cyclosporine, mycophenolate, or tocilizumab. Given the history of FS with clinical and serological evidence of RA activity in this patient, idiopathic ICI-induced neutropenia was deemed to be unlikely. In FS, anti-G-CSF antibodies have been a key proposed mechanism driving neutropenia, the levels of which may have been informative of the underlying immune process in this case but were not readily available at the time. Direct destruction of maturing granulocytes by populations of T or NK(T)-like large granular lymphocytes (LGLs) has also been well-described in FS. While a clonal population of phenotypically abnormal T lymphocytes or LGLs was not found in this patient’s bone marrow, broad immune stimulation by ICI therapy likely explains the polyclonal marrow lymphocytosis, some of which may have contributed to direct cytotoxic or secreted cytokine-induced granulocyte destruction.

ICIs can reactivate pre-existing autoimmune diseases, including rheumatoid conditions, with an estimated 55%–56% risk of flare in patients with pre-existing RA who receive ICI (9, 10). This case highlights the potential for ICIs to trigger life-threatening toxicity, warranting prompt immunosuppressive treatment tailored to the lead differential diagnosis through multidisciplinary management. It also illustrates the potential for recurrent autoimmune processes to follow similar clinical presentations to preceding episodes, with neutropenia being the predominant feature with minimal synovitis in our case. The risk—and to some extent, the type—of irAE can be informed by features of the patient (e.g., demographics and medical history especially any prior autoimmune disease), the tumor (e.g., type and anatomical location), and immunotherapy agent received (e.g., anti-CTLA-4, anti-PD-(L)1, anti-LAG-3, or combination regimens) (11). High-risk patients, such as our case who had a pre-existing autoimmune condition and received a combination immunotherapy regimen, require close monitoring for irAE when commencing ICI. Further mechanistic studies are urgently needed to define the immunologic basis and optimal treatment of all ICI-induced cytopenias, but notably, early recognition and management can restore ANC rapidly despite the profound immune activation of doublet ICI.

## Data Availability

The original contributions presented in the study are included in the article/supplementary material. Further inquiries can be directed to the corresponding author.

## Glossary

ANC: absolute neutrophil count
anti-CCP: anti-cyclic citrullinated peptide
CT: computed tomography
CTLA-4: cytotoxic T lymphocyte-associated protein 4
DMARDs: disease-modifying anti-rheumatic drugs
FS: Felty syndrome
G-CSF: granulocyte colony-stimulating factor
ICIs: immune checkpoint inhibitors
irAEs: immune-related adverse events
IV: intravenous
IVIg: intravenous immunoglobulin
LAG-3: lymphocyte activation gene 3
LGL: large granular lymphocyte
PD-1: programmed cell death-1
RA: rheumatoid arthritis
RF: rheumatoid factor
